# Rambam Hospital is the Birthplace of the Modern Version of Transvaginal Ultrasound

**DOI:** 10.5041/RMMJ.10301

**Published:** 2017-04-28

**Authors:** Ilan E. Timor-Tritsch

**Affiliations:** Director of Obstetrics and Gynecology Ultrasound, Department of Obstetrics and Gynecology, New York University School of Medicine, New York, NY, USA

**Keywords:** History of medicine, transvaginal, ultrasound

## Abstract

The worldwide use of the transvaginal scanning route has revolutionized obstetrical and gynecologic imaging. The long, slow, and at times challenging aspects of its acceptance by the obstetrical and gynecologic community are the subject of this article. From its inception to its recent use, the dedicated doctors in the Department of Obstetrics and Gynecology at Rambam Medical Center, Haifa, Israel, were instrumental in conceiving and then collaborating with an Israeli manufacturer in the construction and worldwide use of the transvaginal ultrasound probe, resulting in the now well-known field of transvaginal sonography.

## BACKGROUND

In 1963, just a few days after completing my medical residency, I began my compulsory three years of military service. I was deployed as a Navy physician and served in the revered “Shayetet 13” unit (comparable to the US Navy Seals and Britain’s Special Boat Service^1^). I was quite proud of my luck; as a member of this unit I would be able to enrich my knowledge of emergency medicine. But in addition, I partook in underwater missions and became familiar with the interesting field of hyperbaric medicine and the physiology of diving—both previously unknown to me. Our unit frequently participated in exercises and real-life combat missions with submariners. It was there that Yoram Bar-Yam, the chief engineer of the submarine *Tanin,* introduced me to Abraham (Ivan) Dror, the (later decorated) captain of the submarine. Yoram and I bonded through the long hours spent together in the narrow spaces of the submarine. In short, we became friends. Although this story may seem unrelated to the topic at hand, its relevance will soon be made clear.

Jumping ahead to 1978, when returning from my fellowship in Cleveland, Ohio on high-risk obstetrics and ultrasound, I began working in the Obstetrics and Gynecology Ultrasound service at Rambam Hospital. There, we used the only available ultrasound scanning route known at the time, the transabdominal route. Transabdominal ultrasound utilizes lower frequencies, yielding clinically satisfactory images of larger structures (e.g. second- and third-trimester fetuses). However, smaller organs such as the ovaries, tiny embryos, and first-trimester fetuses are too far from the transducer tip and cannot clearly be visualized and/or lack sufficient resolution.

The department owned and operated an ultrasound machine manufactured by our “next door” neighbor, the Elscint Company. Along with daily use of a transabdominal transducer probe (which, as mentioned before, enabled imaging through the abdominal wall), the transducer had a small, thin ultrasound probe that functioned at higher sound frequencies—especially constructed for pediatric cardiology and providing high-resolution pictures of the tiny neonatal and infant hearts. I was impressed by the clear images this transducer provided and began to wonder if, somehow, there was a way to insert it into a patient’s vagina—it would be ideal for imaging the female pelvis. The active end of the transducer could be placed in close proximity to the pelvic organs. From there, I theorized that it would generate high-resolution images of the deeply situated ovaries or intrauterine fetuses, as well as hard-to-diagnose pelvic pathologies (e.g. ectopic pregnancies), thereby providing us with a much more detailed visualization of these structures.

## GENERATION OF AN IDEA

I got creative and constructed a makeshift handle: I took two wooden tongue depressors (the ones used by doctors to press down your tongue when examining the throat) and some surgical tape. I then taped the depressors to the back end of the small pediatric transducer to create a more sturdy and longer handle. I then inserted the entire “contraption” into one of the digits of a surgical rubber glove. The result was my “homemade” vaginal transducer. With their permission, I then used this unique transducer on some patients. The resulting pictures were amazingly clear, something we never had before.

**Figure 1:**
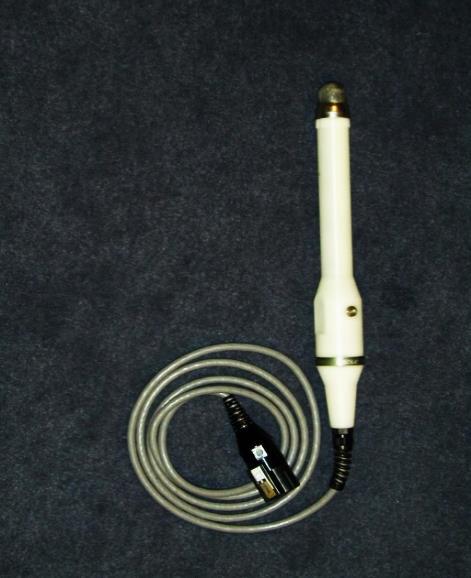
The First Elscint Transvaginal Ultrasound Probe.

Encouraged by the image quality of this “prototype,” I set out to improve it. This is where Yoram, my friend from the submarines, becomes relevant. Yoram had in the meantime accepted an engineering position at Elscint. I turned to him and asked if he could help me by “befriending” the ultrasound manufacturing department, and to see if they would be willing to design and manufacture an elongated casing for the pediatric transducer. In almost no time Yoram handed me the first prototype for my transvaginal ultrasound probe. It was bulky, but it worked. Several months later we placed the prototype “on a diet” and reduced its dimensions; shortly thereafter the second and final version of the transducer became available (Figure 1) and was mass-produced by Elscint, complete with a needle guide attachment for transvaginal puncture procedures.

Although some other companies in the world started to produce and market their own transvaginal transducers, the high resolution of the Elscint probe (analog at that time), provided ultrasound images clearly superior in quality, surpassing even those utilizing the new, digital imaging transducer technology.^2^

## FROM LOCAL TO WORLDWIDE RECOGNITION

All the members of our department used the new ultrasound probe and became quite proficient and experienced in its use; the result was far better diagnoses of female pelvic diseases. Today the obstetrical and gynecologic literature makes it quite clear that it is with thanks to the use of transvaginal sonography that a new chapter was written in evaluating the female pelvis^3^ and fetal anatomy in the first half of pregnancy; this achievement was made in the Department of Obstetrics and Gynecology here at Rambam Medical Center.^4^,^5^ Nevertheless, in the beginning, getting a paper published could by no means be assumed.

Our first article written about use of the transvaginal transducer was submitted to the *Journal of Ultrasound in Medicine* (under the editor George Leopold) and rejected with the comment: “Interesting use of ultrasound, however this is only a descriptive article without data.” However, the exact same article was accepted several weeks later by Frederick Zuspan, then the chief editor of the leading obstetrics and gynecology journal, the *American Journal of Obstetrics and Gynecology*.^2^

The use of the transvaginal probe provided us with the first detailed description of the fetal brain in the first trimester.^6^ We also used this technique for guided puncture procedures of different targets in the pelvis and fetus.^7^ I can name almost all of those who, during those years, worked in our department and contributed both directly and indirectly to developing the concept of evaluating fetal anatomy between 12 and 16 weeks of pregnancy.

Another important contributor to the acceptance of transvaginal ultrasound was Dr Moshe Bronshtein, who promoted this new scanning route and raised it to a whole new level by applying it to imaging first-trimester fetuses; he authored and co-authored more than 30 articles on the subject. One of the first articles that applied the transvaginal scanning route was significant for defining the two kinds of nuchal lesions of the first-trimester fetus.^8^

**Figure 2:**
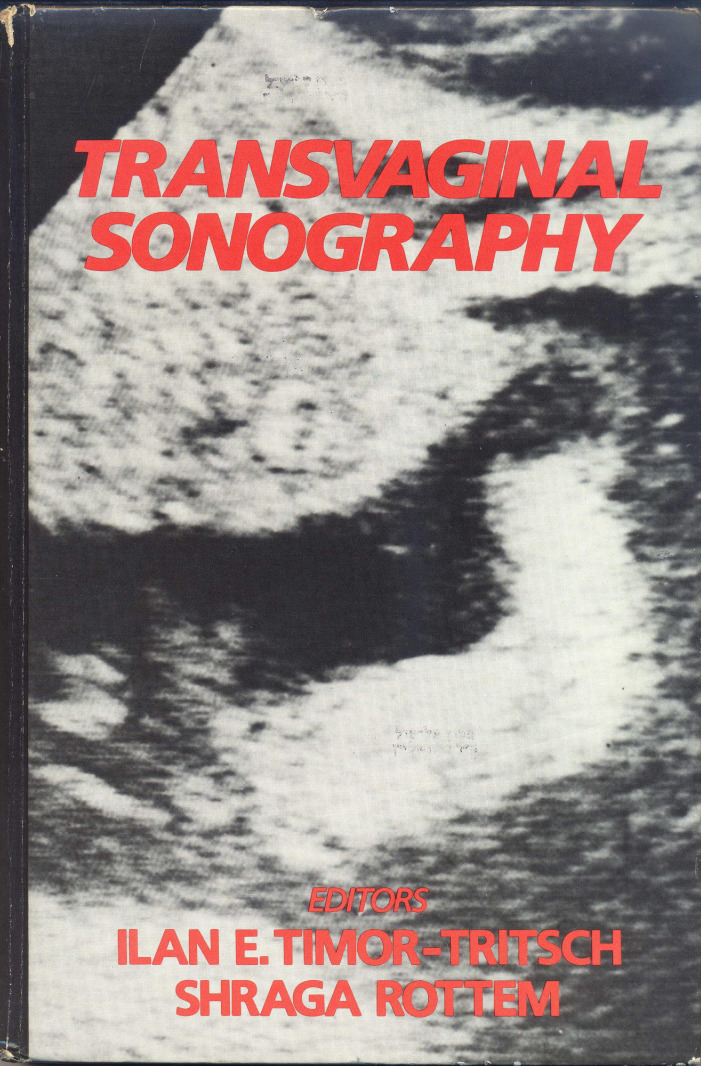
The First Edition of the Book *Transvaginal Ultrasound* by Ilan E. Timor-Tritsch and Shraga Rottem (1987).

**Figure 3:**
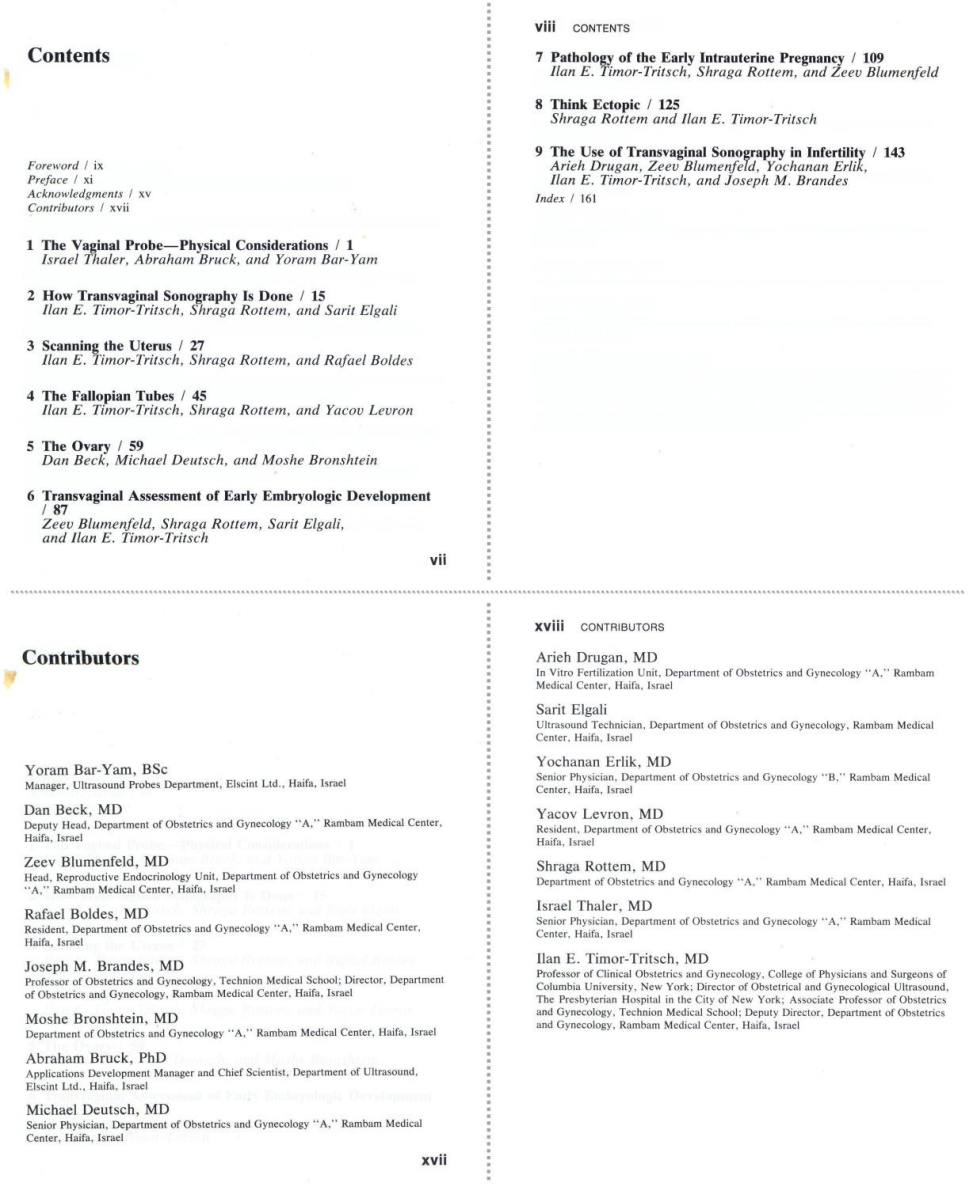
The Contents and Contributors Pages of *Transvaginal Ultrasound* (1987) with the Names of the Co-authors and Members of the Department of Obstetrics and Gynecology at Rambam Hospital.

As this technique caught, I and my colleague, Shraga Rotem, decided to compile more than a dozen of the more noteworthy articles that dealt with the—then—new uses for the transvaginal ultrasound probe. The result was publication of the first book on the subject, entitled *Transvaginal Sonography*^9^ (Figure 2). The book was published in 1987, and each chapter was written by staff of Rambam’s Department of Obstetrics/Gynecology (Figure 3). Encouraged by the interest of obstetricians and gynecologists worldwide in our first edition, we published the second edition in 1991.

Use of the vaginal ultrasound probe enabled us to make a reliable diagnosis of placenta previa, or to rule it out, without endangering the patient or causing bleeding. Our paper on the first use of a transvaginal probe in the above setting was submitted in 1988 to the *British Journal of Obstetrics and Gynecology*. Another rejection of our “first use” awaited us. The letter said (and I quote verbatim): “This article appears to be the first use of transvaginal ultrasound scanning of placenta previa. It is not a substantial report, but can be made brief and it is of general interest. It would stimulate coffee room discussion.” The same article was subsequently accepted in the *American Journal of Obstetrics and Gynecology*.^10^

The new methodology revolutionized prenatal care in other ways. For example, diagnosis of placenta accreta, a life-threatening condition, was usually made based on clinical signs and using abdominal ultrasound transducers, or by taking the pregnant patient to the operating room and preparing her for emergency cesarean delivery with an anesthesiologist at the head of the operating table. If a vaginal, digital examination encountered the presenting placenta, the patient was anesthetized and we proceeded with the cesarean delivery. Transvaginal ultrasound enabled the characterization of placenta accreta and helped in a much simpler and more effective early prediction and diagnosis.^11^

A known expert in obstetric/gynecologic ultrasound, Dr Joseph Woo, wrote a well-researched and documented history of ultrasound use in our specialty, and he underscores some of the facts shared in this brief paper. I quote two entries from Dr Woo’s paper, pertinent to this article:

Moshe Bronshtein, working in Haifa, Israel, described extensively since the early 1990s results of transvaginal sonography in the first trimester.^12^

Ilan E. Timor-Tritsch, working in Israel and later at New York University … systematically studied using high resolution transvaginal transducers in the first trimester, opening up convincingly a new area in fetal ultrasound diagnosis, that of “sono-embryology.”… [He] was also credited for organizing the first three transvaginal ultrasound courses in the United States.^12^

## LOOKING AHEAD

It is now more than 30 years since the introduction of transvaginal ultrasound scanning into the daily practice of obstetrics/gynecology. It is used in the diagnostic process of all aspects of gynecology, including diagnosis of ovarian cancer and ectopic pregnancies. In obstetrics, it enables early detection of first-trimester fetal anomalies. Real-time guidance of transvaginal puncture procedures provides safe access to pelvic organs and to early fetuses. The average obstetrician/gynecologist may not remember the names behind the long and sometimes arduous efforts to popularize the routine use of transvaginal ultrasound. However, combined with the widely practiced transabdominal scanning route, transvaginal ultrasound has finally found its respected and well-earned place in the armamentarium to provide high-level health maintenance of the female patient.

I have great confidence in the future of transvaginal ultrasound at Rambam. I am certain that the tradition of high-quality ultrasound will continue to be maintained, and even further developed in Rambam’s Department of Obstetrics and Gynecology. The department’s newly appointed Director and Chairman, Professor Zeev Weiner, has been deeply interested in the use of ultrasound in our field since he began his career as an obstetrician/gynecologist. This kind of interest can only lead to new and better developments in the use of ultrasound, and I am sure we have not heard the last word on the use of transvaginal ultrasound.
